# Renal Growths

**Published:** 1899-06

**Authors:** 


**Renal Growths.** By T. N. Kelynack, M.D. Pp. xiii., 269. Edinburgh: Young J. Pentland. 1898.

It is just time that this book should appear. A vast amount of periodical literature has now accumulated on the subject, and Dr. Kelynack has given us a careful digest of it, together with his own observations, which, as he is pathologist to the Manchester Infirmary, are likely to be as numerous as those of most men. But the book is not by any means limited to the pathological aspect of the subject; though this naturally occupies the greater part of the book, the clinical aspect is fully considered, and even the operative treatment exhaustively discussed.

At the present time there is a good deal of confusion about the classification of renal tumours—so many varieties are now recognised—and the reader must not expect to gain a perfectly clear knowledge of the subject by the careful perusal of this work. The fact is, the subject has not got beyond the stage of early enquiry; tumours of different forms are gaining recognition as distinct varieties. But the knowledge on the subject which we at present possess is well and clearly given by Dr. Kelynack. The illustrations, both of the naked-eye appearance and of the histology of these renal growths, are remarkably good, and the bibliography is very fully given. We heartily congratulate Dr. Kelynack on the result of the labour which he has obviously bestowed on the production of this work.

